# Long-Term Preservation of Renal Function in Septic Shock Burn Patients Requiring Renal Replacement Therapy for Acute Kidney Injury

**DOI:** 10.3390/jcm10245760

**Published:** 2021-12-09

**Authors:** Filippo Mariano, Consuelo De Biase, Zsuzsanna Hollo, Ilaria Deambrosis, Annalisa Davit, Alberto Mella, Daniela Bergamo, Stefano Maffei, Francesca Rumbolo, Alberto Papaleo, Maurizio Stella, Luigi Biancone

**Affiliations:** 1 Nephrology, Dialysis and Transplantation U, University Hospital City of Science and Health, CTO Hospital, 10126 Torino, Italy; zhollo@cittadellasalute.to.it (Z.H.); alberto.mella@unito.it (A.M.); dbergamo@cittadellasalute.to.it (D.B.); luigi.biancone@unito.it (L.B.); 2 Department of Medical Sciences, University of Torino, 10126 Torino, Italy; consuelo.debiase@gmail.com (C.D.B.); ilaria.deambrosis@unito.it (I.D.); francesca.rumbolo@unito.it (F.R.); 3 Nephrology and Dialysis Unit, Cardinal Massaia Hospital, 14100 Asti, Italy; smaffei@asl.at.it; 4 Laboratory of Nephrology, University Hospital City of Science and Health, Molinette Hospital, 10126 Torino, Italy; 5 Nuclear Medicine Service, Santa Croce Hospital, 12100 Cuneo, Italy; davit.a@ospedale.cuneo.it (A.D.); papaleo.a@ospedale.cuneo.it (A.P.); 6 Clinical Biochemistry Laboratory, University Hospital City of Science and Health, Molinette Hospital, 10126 Torino, Italy; 7 Burn Center and Plastic Surgery, University Hospital City of Science and Health, CTO Hospital, 10126 Torino, Italy; mstella@cittadellasalute.to.it

**Keywords:** acute kidney injury, septic shock, outcome, proteinuria, glomerular filtration rate

## Abstract

Background. The real impact of septic shock-associated acute kidney injury (AKI) on the long-term renal outcome is still debated, and little is known about AKI-burn patients. In a cohort of burn survivors treated by continuous renal replacement therapy (CRRT) and sorbent technology (CPFA-CRRT), we investigated the long-term outcome of glomerular and tubular function. Methods. Out of 211 burn patients undergoing CRRT from 2001 to 2017, 45 survived, 40 completed the clinical follow-up (cumulative observation period 4067 months, median 84 months, IR 44-173), and 30 were alive on 31 December 2020. Besides creatinine and urine albumin, in the 19 patients treated with CPFA-CRRT, we determined the normalized GFR by 99mTc-DTPA (NRI-GFR) and studied glomerular and tubular urine protein markers. Results. At the follow-up endpoint, the median plasma creatinine and urine albumin were 0.99 (0.72–1.19) and 0.0 mg/dL (0.0–0.0), respectively. NRI-GFR was 103.0 mL/min (93.4–115). Four patients were diabetic, and 22/30 presented at least one risk factor for chronic disease (hypertension, dyslipidemia, and overweight). Proteinuria decreased over time, from 0.47 g/day (0.42–0.52) at 6 months to 0.134 g/day (0.09–0.17) at follow-up endpoint. Proteinuria positively correlated with the peak of plasma creatinine (*r* 0.6953, *p* 0.006) and the number of CRRT days (*r* 0.5650, *p* 0.035) during AKI course, and negatively with NRI–GFR (*r* −0.5545, *p* 0.049). In seven patients, urine protein profile showed a significant increase of glomerular marker albumin and glomerular/tubular index. Conclusions. Burn patients who experienced septic shock and AKI treated with CRRT had a long-term expectation of preserved renal function. However, these patients were more predisposed to microalbuminuria, diabetes, and the presence of risk factors for intercurrent comorbidities and chronic renal disease.

## 1. Introduction

Acute kidney injury (AKI) sustained by septic shock is a severe complication in critically ill patients, influencing patient survival and kidney functions in the long term.

Observational studies demonstrated a significant risk of deterioration of renal function in patients that survived an episode of AKI, even if a “renal recovery”, as determined by the return of eGFR to pre-morbid levels, occurred. In patients without pre-existing renal dysfunction, aging, co-morbidities, and renal function at discharge were the main determinants of the long-term functional outcome [1,2]. These observations led to a discussion on the causal relationship between AKI and CKD, and on the role of preexisting comorbidities such as diabetes, hypertension, cardiovascular disease, and preexisting CKD [1,2,3,4]. Furthermore, the AKI survivors are a heterogeneous population, and AKI etiology largely varies. Therefore, characterizing and identifying selected individuals with the highest risk for chronic renal disease is of great importance [2,3,4].

Among critically ill patients, severe burn patients can be considered a good study model for septic-shock-associated AKI, given that they are a homogeneous population for which the initial insult can be quantified and the septic process constantly develops and lasts for several weeks. Mortality worsens when renal replacement therapy (RRT) is required; a recent monocentric study reported a mortality rate as high as 81.5% in a population of 216 burn patients managed with RRT over 7 years [3].

Beyond AKI, proteinuria is a common clinical finding in burn patients with septic-shock-associated AKI [5,6,7]. Filtered break-down products, exotoxins, and/or the massive production and release of a wide array of endogenous, circulating pro- and anti-inflammatory molecules are thought to be involved. These active molecules can affect hemodynamics and exert a deleterious effect on the kidney by inducing increased glomerular permeability and proteinuria [7,8,9,10].

Studies evaluating the long-term outcome of septic-shock-associated AKI requiring RRT are few and not conclusive. In burn patients, the majority of these studies have a short follow-up or restrained to hospital stay time [5,11,12,13,14,15]. In one such study with a follow-up extended up to 1 year, patients with AKI showed an increased incidence of severe CKD and necessity of chronic dialysis (4.58% in burn patients with AKI vs. 0.33% without AKI) [16]. Similarly, in a Finnish registry study, the risk of dialysis during the postburn period was increased compared with the general population (OR 2.40, CI 1.73–3.23) [4]. On the other side, in another recent registry study involving 32 survivor burn patients, the need for RRT subsided before discharge, and patients rarely needed long-term RRT [17]. However, no study thus far has evaluated the finer alterations of glomerular and tubular function years later in burn patients who suffered from a severe form of septic-shock-associated AKI.

Our study aims to investigate the long-term renal outcome of 30 severe burn survivors who experienced septic shock, multi-organ failure, and AKI treated with CRRT by determining glomerular filtration rate and studying glomerular and tubular marker proteins.

## 2. Materials and Methods

### 2.1. Study Design and In-Hospital AKI Management

**Figure 1:**
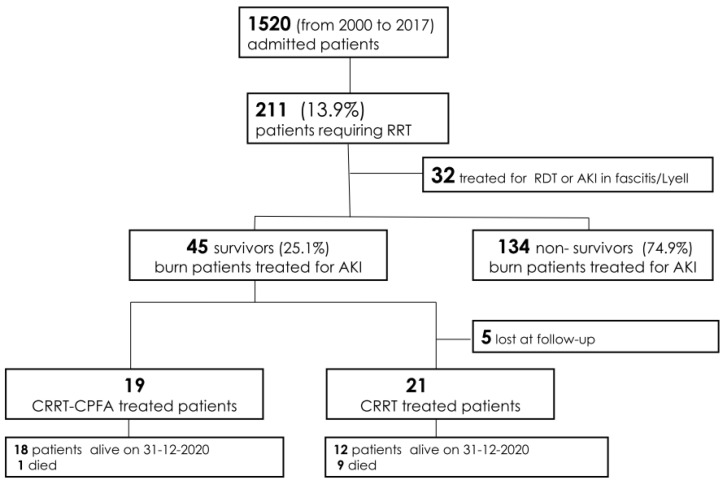
Flow-chart of the study. Abbreviations: RRT = renal replacement therapy; RDT = regular dialysis treatment; AKI = acute kideny injury; CRRT = continuous renal replacement therapy; CRRT-CPFA = CRRT-coupled plasma filtration adsorption.

From January 2001 to December 2017, 1520 adult patients were admitted to the Burn Center at CTO Hospital. Out of 1520 patients, 211 underwent CRRT, and 179 of these cases were due to severe AKI. The survival rate was 25.1% (45 patients), and among these patients, 26 were only CRRT-treated (CRRT patients) [15], and an additional 19 were treated with sorbent therapy (CPFA-CRRT patients) as part of their septic shock treatment (Figure 1) [18,19,20].

The study was conducted according to the Helsinki Declaration and approved by the Ethics Committee of AOU Citta’ della Salute e Scienza di Torino (dossier n. CS2/908).

Informed consent to the proposed treatments, retrospective review of the medical notes, and analysis of the collected data was obtained from the patients or substitute decision-maker.

### 2.2. In-Hospital Burn and AKI Management

Systemic treatment of burn patients, diagnosis, and surgical procedures, including the treatment of septic shock, were managed by a multidisciplinary team based on current guidelines [21].

CRRT was started when patients were in a trend of fluid overload that was not responsive to conservative management, including maximal diuretic therapy, and oliguria, severe hyperkaliemia, severe acidosis, or uremic complications. CPFA-CRRT was chosen as an additional therapy to CRRT when: (1) the patients were in septic shock with AKI-RRT and multiorgan failure, and; (2) microbiological confirmation of the septic event through blood cultures was positive for multidrug-resistant strains, and; (3) there was no clinical response after 72 h of appropriate antibiotic treatment [19,20].

### 2.3. Technical Aspects of CRRT and CPFA-CRRT Treatment

CRRT procedures were carried out with a multifiltrate apparatus (Fresenius Medical Care AG, Bad Homburg, Germany), equipped with a high flux polysulfone filter (AV1000, Fresenius Medical Care) at a blood flow rate of 120 mL/min. Fluid infusion and dialysate flow rates were used to accomplish the dialysis target of 20–25 mL/Kg/die [15].

CPFA-CRRT was performed with a dedicated machine that the manufacturer updated over time (Multimat B.IC/Lynda/Amplya, Bellco spa, Mirandola, Italy). CPFA was always carried out by using a polyethersulfone plasma filter (0.5 m^2^, MPS 05, Bellco) placed in series with a highly permeable polyethersulfone hemodialyzer (1.4 m^2^, BLS814G, Bellco). Plasmafiltrate was adsorbed on an unselective hydrophobic resin cartridge (140 mL for 70 g, with a surface of about 700 m^2^/g) [18,19,20].

According to CPFA protocol, blood flow rate ranging from 100 to 180 mL/min and effluent rates were set by the target of dialysis adequacy. We set an exchange of 3–4 L/h of effluent at the start to accomplish the dialysis target of 20–25 mL/Kg/die [20]. According to the manufacturer’s protocol, plasma filtration rate was maintained between 15–25% of blood flow rate.

The vascular access of CPFA was provided by 12 F double lumen venous catheter inserted in the jugular or femoral vein.

In order to provide CRRT and CPFA-CRRT effectively and safely, either unfractionated heparin or citrate were used for anticoagulation of the extracorporeal circuit following our previous experiences [15,18,19] and, when available, the manufacturer’s instructions.

The choice of heparin or citrate was done on the basis of the patients’ characteristics. Specifically, the regional citrate anticoagulation (RCA) strategy was the first choice when the patient presented (1) a high bleeding risk (defined as bleeding alert in the insertion site catheter, at the tracheostomy, the gastrointestinal tract, or in surgical wounds), or (2) frank bleeding necessitating transfusion of packed red blood cells [13,17,18]. In all patients undergoing RCA, dialysate was Ca++-free, and a commercial 10% calcium chloride solution was infused in a separate line at the end of the venous circuit [18,19,20].

Heparin was administered pre-filter and standardized to an initial bolus of 1250 U followed by 1000 U/h. Subsequent adjustments were made accordingly to obtain coagulation parameters of PTT > 60 s.

### 2.4. Nephrological Follow-Up after Discharge

The 45 surviving patients entered a program of nephrological follow-up, which included scheduled visits, determination of creatinine, urine examination, and proteinuria at months 1, 2, 3, and 6 after the burn injury. Thereafter, the patients were seen as outpatients at months 9 and 18, and every 4–6 years or sooner if they felt any change in their status.

From January 2020, for all 18 surviving CPFA-CRRT patients, a new visit was scheduled to evaluate their clinical conditions (occurred comorbidities, ongoing therapy) and to study renal function by urine marker proteins and by determination of glomerular filtration rate (Figure 1).

At the same time, we contacted the 26 CRRT patients treated from January 2001 to December 2017. Of these, 5 were lost to follow-up, 9 died, and 12 were alive on 31 December 2020 (Figure 1).

### 2.5. Study of Urine Protein Profile and Determination of Normalized Radio Isotopic GFR (NRI–GFR)

Urine protein profile was done by electrophoresis and nephelometric quantification of specific glomerular (albumin, transferrin, immunoglobulin G, alpha2-macroglobulin) and tubular (retinol-binding protein, alpha1-microglobulin) marker proteins. The type of proteinuria (glomerular, tubular, or mixed glomerular-tubular) was determined by calculating the glomerular-tubular ratio of protein markers (MDI-LABLINK software) [22,23]. Urinary sediment was carried out on a spot urine sample.

Determination of NRI–GFR was done in patients fasted for 8 h and adequately hydrated before the injection of the radioisotope. After administration of 37 MBq of 99 mTc-DTPA, four samples of EDTA-anticoagulated blood (2 at 90 min and 2 at 180 min) were collected. The standard, simulating the plasma pool, was prepared with 18 MBq 99 mTc-DTPA in 1 L of water. Duplicated blood and standard samples were measured in a well scintillation counter. The relative counting error was <2.0%.

The total plasma clearance was determined according to the Russel method. Values of glomerular filtration rate were normalized to the body size using BSA (NRI-GFR, mL/min) according to the BNMS’s guidelines [24].

### 2.6. Statistical Analysis

Continuous data are expressed as median with quartiles (the 25th and 75th percentiles) and categorical data as frequencies and percentages.

Student’s *t*-test, Fisher’s exact test, or ANOVA with multicomparison Newman–Keuls test and linear regression analysis were used when appropriate.

*p* < 0.05 value was considered statistically significant. Statistical computing and graphics were performed by Statistica v.10.1 (Statsoft, Tulsa, OK, USA).

## 3. Results

### 3.1. Baseline Characteristics of Patients and Long-Term Outcome of Creatinine and Urine Albumin

The basal characteristics of the 40 patients enrolled in the follow-up are shown in Table 1. Baux index was similar for CRRT and CPFA-CRRT patients at the time of AKI, while age was significantly higher and TBSA lower for CRRT patients than for CPFA-CRRT patients. Mortality was significantly higher for CRRT patients (43% vs. 5.3%), and out of all 40 patients, 30 were alive at the follow-up endpoint on 31 December 2020 (Table 1).

**Figure 2:**
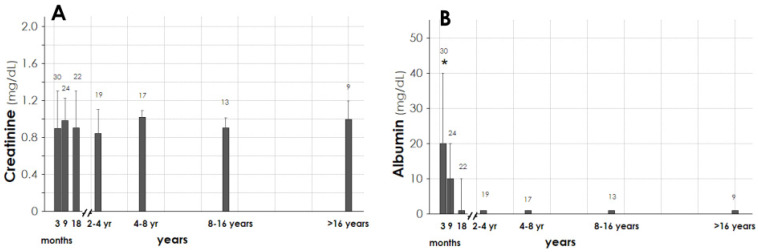
Outcome of plasma creatinine (panel **A**) and urine albumin (panel **B**) for patients alive on 31 December 2020. Panel **A** shows that plasma creatinine was in normal range over the long-term follow-up. Panel B shows that urine albumin markedly decreased within the first year, and then disappeared. Data are given as median (interquartile 1–3), * *p* < 0.05.

The patients were followed for a cumulative time of 338.9 years (4067 months), with a median value of 84 months/patient (44–173) (Table 1). Over the follow-up, the plasma level of creatinine was stable (Figure 2, panel A), with a median value of 0.94 mg/dL (0.72–1.03).

Urine albumin was detectable in significant amount after the burn injury, then markedly decreased within the first year, disappeared 2–4 years later, and was undetectable at follow-up endpoint (Figure 2, panel B).

### 3.2. Risk Factors for the Development of CKD

**Figure 3:**
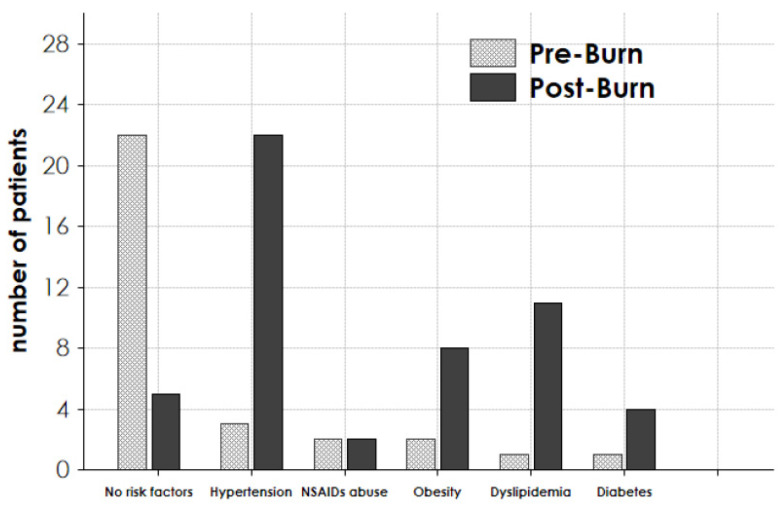
Presence of risk factors for chronic kidney disease in the 30 considered patients. The presence of the specified risk factor was evaluated for each patient in pre-burn period (Pre-Burn) and at the follow-up end, on 31 December 2020 (Post-Burn).

Among the patients alive on 31 December 2020, 22/30 subjects presented risk factors for the future development of CKD, whereas at the time of the burn injury, only 5/30 presented risk factors (Figure 3, first left columns). Indeed, on 31 December 2020, 22 patients were hypertensive, 8 were obese/overweight, 11 were dyslipidemic, and 4 were diabetic. All hypertensive patients were taking ACE/ARB as an anti-hypertensive drug (Figure 3).

### 3.3. Study of Long-Term Glomerular Function and Glomerular/Tubular Protein Markers in CPFA-CRRT Patients

**Figure 4:**
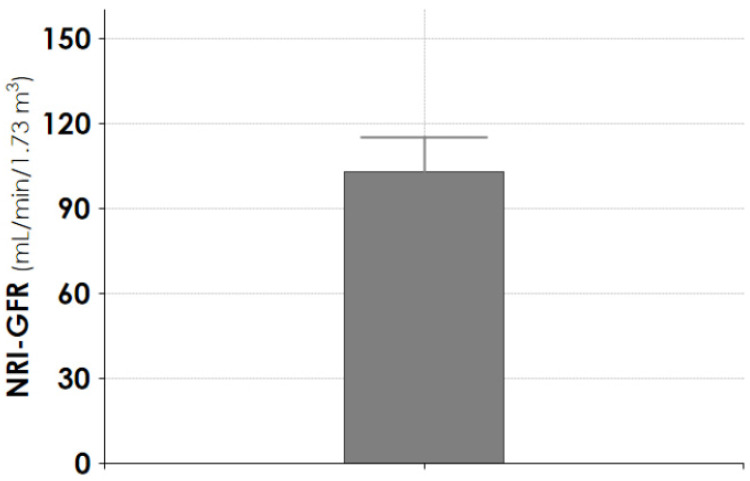
Determination of NRI-GFR at the follow-up endpoint in patients treated with CPFA-CRRT. Data are given as median (interquartile 1–3). NRI-GFR = normalized radio isotopic glomerular filtration rate.

At the follow-up endpoint, the median plasma creatinine was 0.89 mg/dL (0.51–1.3), and the median 24 h proteinuria was 0.134 gr/day (0.09–0.17). The determination of glomerular filtration rate by radioisotope technique showed a median value of 103.0 mL/min (93.4–115.0) (Figure 4).

**Figure 5:**
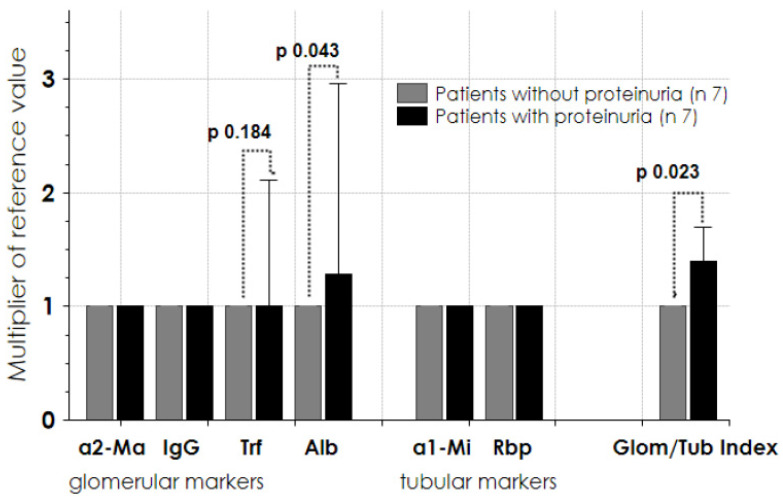
Glomerular and tubular protein markers at follow-up endpoint (14 patients). Seven patients had normal values of protein markers (grey column) and 7 patients presented significantly high concentrations of protein markers (black column) (see ref. [22]). Data are given as median (interquartile 1–3). a2Ma = alpha2-macroglobulin; IgG = immunoglobulin G; Trf = transferrin; Alb = albumin; a1-Mi = alpha1-microglobulin; Rbp = retinol binding protein; Glom/Tub index = glomerular/tubular index.

We evaluated the urine protein profile in 14 patients. As shown in Figure 5, no significant amount of glomerular/tubular protein markers was found in seven patients (grey columns). In the remaining seven patients (black columns), there was an increase of glomerular markers of low molecular weight, such as albumin (*p* 0.043) and transferrin (*p* 0.184), with a significantly higher glomerular/tubular index. All seven of these patients suffered from hypertension and were taking ACE/ARB as an anti-hypertensive drug; one was affected by diabetes, three by dyslipidemia, one by obesity, and two were overweight.

In all studied patients, the tubular marker proteins were in the normal range (Figure 5, central block columns).

### 3.4. Relationship between Proteinuria, AKI Severity, and NRI-GFR in CPFA-CRRT Patients

**Figure 6:**
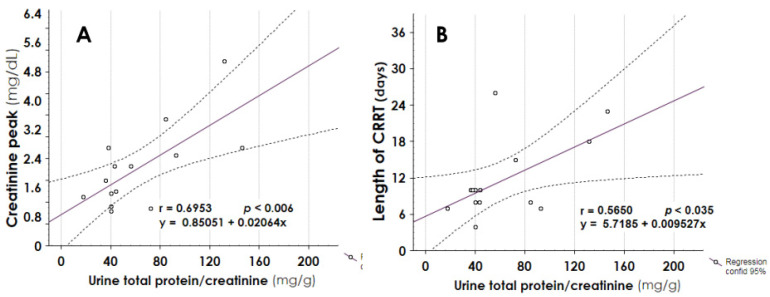
Correlation between urine total protein at follow-up endpoint and creatinine peak during burn injury (panel **A**), or days of CRRT (panel **B**). A significant positive correlation was found between proteinuria and the 2 indexes. CRRT = Continuous Renal Replacement Therapy.

Urine total protein/creatinine ratio at the last follow-up endpoint significantly correlated with creatinine peak during burn injury (Figure 6, panel A, *r* 0.6953, *p* 0.006) and with days of CRRT (Figure 6, panel B, *r* 0.5650, *p* 0.035).

**Figure 7:**
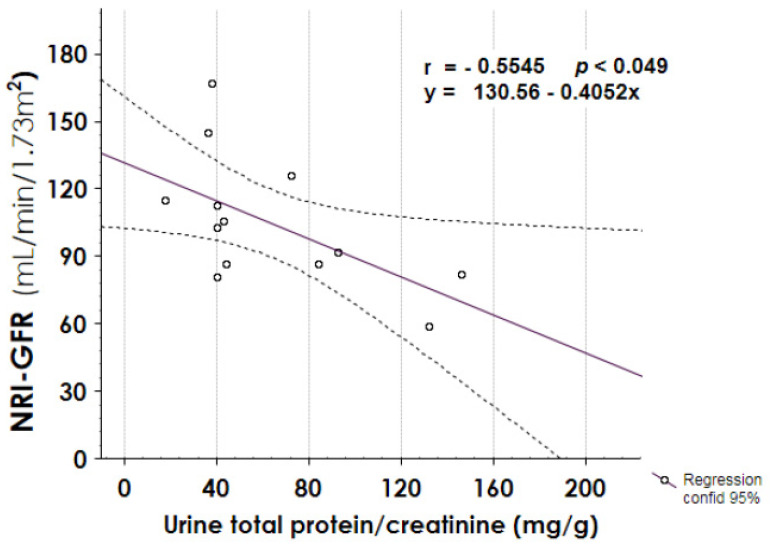
Correlation between urine total protein at follow-up endpoint and normalized radio-isotopic GFR (NRI-GFR), or follow-up length. A negative correlation was found between proteinuria and NRI-GFR.

In addition, urine total protein/creatinine ratio negatively correlated with NRI–GFR value (Figure 7, panel A, *r* −0.5546, *p* 0.049), and it showed a positive trend of correlation with the months of follow-up (Figure 7, panel B, *r* 0.4465, *p* 0.109).

## 4. Discussion

Burn patients suffering from septic shock and AKI treated with CRRT who had a full recovery of renal function within 3 months showed a long-term high incidence of microalbuminuria and hypertension, with a stable conserved glomerular function.

In recent years, many studies demonstrated a strong association between AKI episodes and the development of chronic renal damage, identifying as risk factors some baseline characteristics, such as advanced age, diabetes mellitus, decreased baseline eGFR, low serum albumin concentration, APACHE II score, and elevated IL-6 concentration [1,2,25,26]. However, the severity of AKI expressed as the magnitude of peak in serum creatinine, RRT requirement and duration, time and extent of recovery, and the number of AKI episodes were also found to play a significant role [1,27,28]. Therefore, while some patients fully recover, when the severity of the initial AKI reaches a certain threshold, its course can be altered leading to chronic, progressive disease.

Currently, we know that AKI can arise from different causes, which can influence the long-term outcome. We chose to evaluate the long-term outcome in burn patients with septic shock and AKI requiring CRRT. Studies in the general burn population showed that kidney function usually recovered during the hospital stay [5,11,12,13,14,15,16,29,30,31,32]. However, a significant risk of renal function deterioration at one-year follow-up was described [16], whereas in registry studies the need for RRT was rare [17,18]. In our cohort of patients who recovered their renal function within 3 months, after the median follow-up of 8.8 years (extended up to more than 16 years for nine patients), the creatinine plasma levels were in the normal range (Figure 2). Similarly, in the case series of patients treated with CPFA-CRRT, for whom we determined the renal filtration by radioisotope technique, the median value of GFR was 103 mL/min. According to what was described for AKI patients with similar baseline characteristics [21,28,31], the excellent long-term preserved renal function can be suggested by the full recovery of eGFR after one year, given that the patient population was relatively young (median 52 years at AKI episode), healthy, and had few baseline risk factors for CKD (Figure 7). During the follow-up, the mortality in group CRRT was 35%, significantly higher than the 5% seen in group CPFA–CRRT (Table 1). In effect, at the follow-up endpoint, the median age was 74 and 52 years for CRRT and CPFA-CRRT patients, respectively, with a significant difference of 22 years. However, the risk of burn death was the same between the two groups, as the CRRT group was affected by a significantly lower TBSA% (Table 1). It is conceivable that this great age difference deeply impacted the long-term survival, independently from any reason related to early death risk after the burn injury.

However, on 31 December 2020, at the median age of 63 years, most of the 30 patients presented several risk factors for CKD: 22 patients (73.3%) were hypertensive, 8 were overweight, 11 were dyslipidemic, and 4 were diabetic. AKI is, per se, a risk factor for long-term blood pressure elevation [32], and it has also been proven that burns can cause alterations of organ function with a prolonged inflammatory and hypermetabolic state [33]. Therefore, it is conceivable that both AKI and burns contributed to the long-term development of hypertension, dyslipidemia, diabetes, and overweight, which in turn could promote CKD onset [34].

In this study, we also evaluated the outcome of proteinuria. Proteinuria is a constant feature of sepsis- and burn-associated acute kidney injury [6,7,35,36,37,38], it appears shortly, and it is detectable for several weeks until the patient is critically ill [7]. Sepsis, TBSA > 30%, and age were shown to be the main independent risk factors for proteinuria [35,36,37], whose length and entity directly correlated with the severity of burn and septic process [6,35,36,37,38,39,40]. It has been shown that the plasma of septic shock burn patients with AKI is able to increase permeability to albumin, decrease expression of nephrin on podocytes in culture [39], and exert a pro-apoptotic effect on podocytes and tubular cells [39,40]. However, in our patients, proteinuria disappeared within one year.

At the follow-up endpoint, we determined the urine protein profile and found that urine albumin and the derived glomerular-tubular index were significantly increased in half of the patients (Figure 5), demonstrating the presence of selective glomerular proteinuria. Alpha1–microglobulin and retinol-binding proteins were in the normal range in all patients, demonstrating the integrity of tubular function. An increased glomerular basement membrane permeability to albumin in terms of microalbuminuria was commonly observed in post-AKI episodes [41] and in patients with hypertension [2,32]. These observations suggest the absence of any structural tubular and interstitial alterations in these burn patients years later, such as shown in the progression of chronic renal diseases when the tubular adsorption of smaller proteins is disturbed [22,23].

Searching for a relationship between the initial injury and proteinuria, we found a significant positive correlation between proteinuria at follow-up end and the number of days of CRRT, and the creatinine peak during AKI episode (Figure 6). These data can suggest that the risk of long-term proteinuria was related to the severity of septic AKI. However, the paucity of the data did not allow us to link the long-term alteration in glomerular permeability to the severity of initial burn injury. Other factors could be involved in the pathogenesis of proteinuria; most of the proteinuric patients were hypertensive and taking ACE/ARB as anti-hypertensive drugs, four were affected by diabetes, three by dyslipidemia, one by obesity, and two were overweight. In addition, proteinuria showed a positive trend of correlation with the length of follow-up (*p* 0.109) and correlated negatively with NRI-GFR. Therefore, it seems that the patients who suffered from septic AKI could have a reduction of their renal functional reserve, proportional to kidney injury extent and the length of follow-up and thus were more predisposed to the intercurrent comorbidities acting as determinant factors of further kidney damage.

Our study suffers from some limitations. First, it is a single-center study that enrolled only 40 patients, justified by the high mortality rate in this setting, especially in those requiring RRT. Second, not all data were available for all patients, as some of them were not easy to reach for organizing visits; negative psychological consequences due to burn injury have been widely reported, showing persistence of psychosocial burden over time after burn and, sometimes, refusal of contact with the center of care [42]. Third, it is not easy to rule out other potential factors over the long-term follow-up beyond septic-shock-associated AKI, such as intercurrent infections or pharmacological treatments that might have played a role in the final GFR and urine protein profile.

Notwithstanding these limitations, our study presents several points of strength. First, to the best of our knowledge, this is the first study after septic-shock-associated AKI evaluating long-term glomerular and tubular integrity by NRI-GFR and urine protein markers. Second, we studied a homogeneous population, treated in the same center with a defined protocol of dialytic technique for septic-shock-associated AKI. This study can thus stand out in a “mare magnum” of large randomized controlled trials where the co-existence of very heterogeneous patient populations does not allow the detection of the finest alterations in renal function [43]. Third, our study is based on sequential clinical examination with reliable data on renal function collected for a very long time, more than 16 years in nine patients.

## 5. Conclusions

Septic shock burn patients treated with CRRT for AKI had a long-term good expectation of conserved renal function, but they were more predisposed to kidney damage by intercurrent comorbidities. Even if further studies are needed to confirm these results, this should encourage a nephrological follow-up to recognize and correct the risk factors for potential CKD development.

## Figures and Tables

**Table 1:** Baseline characteristics of 40 survivor burn patients treated with CRRT (*n* 21) or CPFA-CRRT (*n* 19) enrolled in the follow-up.

	All RRT Patients	CRRT Patients	CRRT-CFFA Patients	*p*
Patients (*n*)	40	21	19	-
Cumulative follow-up time (months)	4067	1728	2339	-
Gender ratio (male/female)	29/11	13/8	16/3	0.11
Age (years)	52.0 (43.2–68.0)	67.0 (53.0–74.0)	46.0 (30.0–51.0)	0.01
Age at follow-up end (years)	63.0 (49.0–73.0)	74.0 (59.6–81.0)	52.0 (44.3–62.0)	0.01
Follow-up time (median, months)	84 (44–173)	54 (36–159)	101 (68–206)	0.07
Mortality (%, *n*)	25.0%, 10	43.0%, 9	5.3%, 1	0.02
Total body surface area (%)	37.5 (22.5–50.0)	30.0 (20.0–40.0)	45.0 (35.0–60.0)	0.01
Baux index	0.32 (0.16–0.62)	0.43 (0.13–0.61)	0.30 (0.19–0.63)	0.70
Septic shock (%, *n*)	95.0%, 38	95.0%, 19	100%, 19	0.35
Mechanical ventilation (%, *n*)	97.5%, 39	95.2 %, 20	100%, 19	0.54
SOFA score (at 1st day of treatment)	10 (9–12.5)	10 (9–12)	10 (9–13)	0.73
CRRT/CPFA-CRRT duration (days)	9.0 (5.50–21.5)	8.0 (5.0–29.0)	10.0 (7.0–20.0)	0.58
Citrate anticoagulation (%, *n*)	70.0%, 28	80.9%, 17	57.8%, 11	0.08
Plasma creatinine (at the start, mg/dL)	2.2 (1.3–3.0)	2.4 (2.2–3.1)	1.4 (0.9–2.2)	0.01
Plasma creatinine (at the end, mg/dL)	1.3 (0.9–1.8)	1.3 (1.1–1.9)	1.1 (0.7–1.7)	0.06
Plasma creatinine (at the peak, mg/dL)	2.4 (1.4–3.1)	2.9 (2.2–3.4)	1.8 (1.1–2.7)	0.01

Data are given as median (the 25th and 75th percentiles) or as percentage when appropriate. RRT = renal replacement therapy; CRRT = continuous renal replacement therapy; CRRT-CPFA = Coupled plasma filtration adsorption with continuous renal replacement therapy.

## Data Availability

The datasets used and/or analyzed during the current study are available from the corresponding author on reasonable request.
